# Optimal Dietary Intake Composition of Choline and Betaine Is Associated with Minimized Visceral Obesity-Related Hepatic Steatosis in a Case-Control Study

**DOI:** 10.3390/nu14020261

**Published:** 2022-01-08

**Authors:** Ting-Yu Chang, Chien-Hsien Wu, Chi-Yang Chang, Fu-Jen Lee, Bei-Wen Wang, Jia-Yau Doong, Yu-Shun Lin, Chang-Sheng Kuo, Rwei-Fen S. Huang

**Affiliations:** 1 Department of Nutritional Science, Fu Jen Catholic University, New Taipei City 242062, Taiwan; tingyoyo871123@gmail.com (T.-Y.C.); 141600@mail.fju.edu.tw (J.-Y.D.); fj03899@gapp.fju.edu.tw (Y.-S.L.); 2 Ph.D. Program in Nutrition and Food Science, Fu Jen Catholic University, New Taipei City 242062, Taiwan; jasper5j@yahoo.com.tw; 3 Department of Gastroenterology and Hepatology, Taipei Hospital, Ministry of Health and Welfare, New Taipei City 242, Taiwan; 4 Department of Gastroenterology and Hepatology, Fu Jen Catholic University Hospital, New Taipei City 243089, Taiwan; chiyang1112@gmail.com (C.-Y.C.); paul6130@gmail.com (F.-J.L.); 5 Department of Nutrition, Fu Jen Catholic University Hospital, New Taipei City 243089, Taiwan; a8307142001@gmail.com

**Keywords:** methyl donor nutrients intake, choline, betaine, folate, obesity, hepatic steatosis

## Abstract

Few studies on humans have comprehensively evaluated the intake composition of methyl-donor nutrients (MDNs: choline, betaine, and folate) in relation to visceral obesity (VOB)-related hepatic steatosis (HS), the hallmark of non-alcoholic fatty liver diseases. In this case–control study, we recruited 105 patients with HS and 104 without HS (controls). HS was diagnosed through ultrasound examination. VOB was measured using a whole-body analyzer. MDN intake was assessed using a validated quantitative food frequency questionnaire. After adjustment for multiple HS risk factors, total choline intake was the most significant dietary determinant of HS in patients with VOB (Beta: −0.41, *p* = 0.01). Low intake of choline (<6.9 mg/kg body weight), betaine (<3.1 mg/kg body weight), and folate (<8.8 μg/kg body weight) predicted increased odds ratios (ORs) of VOB-related HS (choline: OR: 22, 95% confidence interval [CI]: 6.5–80; betaine: OR: 14, 95% CI: 4.4–50; and folate: OR: 19, 95% CI: 5.2–74). Combined high intake of choline and betaine, but not folate, was associated with an 81% reduction in VOB-related HS (OR: 0.19, 95% CI: 0.05–0.69). Our data suggest that the optimal intake of choline and betaine can minimize the risk of VOB-related HS in a threshold-dependent manner.

## 1. Introduction

Non-alcoholic fatty liver disease (NAFLD) is the most common chronic liver disease, affecting a quarter of the population in Western countries and Asia [1]. The severity of intrahepatic fat accumulation in NAFLD is related with an increase in oxidative stress, proinflammation status, and chronic kidney disease complications [2,3], all of which are strongly linked to overweight and obesity (OB) [4]. OB is considered the main driver of NAFLD-associated peripheral and hepatic metabolic dysfunction [4]. Hepatic steatosis (HS), the hallmark of NAFLD, is associated with a 60–90% prevalence among obese patients [5]. In particular, visceral obesity (VOB) is associated with a higher risk of type 2 diabetes mellitus, dyslipidemia, and insulin resistance [6] and is more associated with HS severity and NAFLD-related mortality across ethnicities [7]. Dietary modification to reduce excess adiposity can help reduce OB-related HS and the risks of progression to NAFLD and associated mortality [8].

Methyl-donor nutrients (MDNs), including choline, betaine, and folate, regulate hepatic lipid homeostasis, energy metabolism, adiposity, and related metabolic disorders of dyslipidemia and insulin resistance [9]. De novo synthesis of the choline moiety derived from three methyl groups occurs through folate- and betaine-mediated methylation pathways to form phosphatidylcholine (PtCh), which has the lipotropic action of secreting hepatic triglyceride as very-low-density lipoprotein [10]. Two thirds of dietary choline is used for de novo PtCh synthesis, but significant amounts are oxidized to betaine to spare folate for methyl donors, which is critical for epigenetic regulation of lipid and bioenergetic metabolizing enzymes [11,12]. High-fat diet–induced obese mice when fed a choline-deficient diet exhibited hypermetabolism, weight loss [13], and exacerbated fatty liver development [14]. Genetic polymorphism of phosphatidylethanolamine N-methyltransferase (PEMT) which, essentially, contributes to the secretion of very low-density lipoprotein, has been associated with increased risk of NAFLD [15]. Deletion of betaine–homocysteine methyltransferase (BHMT) for methyl donor generation has been associated with disturbed hepatic MDN homeostasis, impaired lipid synthesis, and caused fatty liver and hepatocellular carcinoma in mice [16]. Steatosis in folate-deficient mice was observed to increase betaine and choline demand for phospholipid synthesis and hepatic triglyceride secretion [17]. Dietary supplementation with choline or betaine in high-fat diet–induced obese mice can prevent fatty liver and improve dyslipidemia and insulin resistance [18,19]. Multiple MDN supplementation in mice with diet-induced obesity can also prevent HS progression [20]. Thus, MDN intake plays a vital role in obesity-related HS development.

Limited research has been conducted on the relationship between MDN intake and obesity-related HS risk. Moreover, results on individual MDN effects have been inconsistent. In a large population-based study involving 56,000 Chinese adults, high choline intake was associated with reduced NAFL risks in normal-weight women but not overweight or obese men or women [21]. In 644 patients with NAFLD, low choline intake was associated with the progression of steatosis to fibrosis in postmenopausal women but not with hepatic lipid accumulation [22]. In one study, low serum folate was associated with decreased HS incidence in obese patients; however, the researchers did not assess the dietary folate and other MDNs intake [23]. Higher serum betaine was associated with favorable body fat distribution in Chinese adults [24], whereas high betaine intake by supplementation did not improve visceral and subcutaneous adiposity in Asian males with mild fatty liver [25]. Such a mixed effect of individual MDN status on body fat distribution and HS may be in part due to the lack of consideration for metabolic crosstalk of MDNs and their mutual effect on hepatic lipid and energy homeostasis [10,11,12,26]. No study has explored the association of MDN intake composition with obesity-related HS risks.

Here, we elucidated the relationship between MDNs intake and obesity-related HS by identifying the optimal MDN intake composition that can minimize obesity-related HS risk in a high-risk population with metabolic disorders.

## 2. Materials and Methods

### 2.1. Study Participants

Between January 2020 and March 2021, we recruited participants from the Department of Gastroenterology and Hepatology at the Ministry of Health and Welfare-affiliated Taipei Hospital (TH) and at Fu Jen Catholic University Hospital (FJCUH), New Taipei City, Taiwan. Patients with an ultrasound diagnosis of HS were eligible for inclusion. We excluded patients with hepatitis B or C virus infection, severe cardiovascular disease, chronic kidney disease, neuronal and gastrointestinal disorders, cancer, and weekly alcohol intake >100 g or unknown alcohol intake. Of 111 eligible cases, six were unable to donate extra blood samples and were excluded. In total, we included 105 patients with HS and 104 sex-matched controls, who were patients without HS (confirmed by physicians) with a similar geographical background to the cases. Both cases and controls were interviewed by trained professionals for a complete medical and dietary history. Fasting blood samples were collected from all participants for biochemical measurements. The study protocol was approved by the Joint Ethical Committee at FJUH (IRB-C108075) and TH (IRB-0019-0021). Written informed consent was obtained from all participants.

### 2.2. Ultrasound Diagnosis of Hepatic Steatosis (HS)

The presence of HS was assessed by abdominal ultrasound scanner (ARIETTA60, Hitachi, Tokyo, Japan) by experienced physicians. Steatosis is graded as follows: absent (score 0) when the echotexture of the liver is normal; mild (score 1), when there is a slight and diffuse increase of liver echogenicity with normal visualization of the diaphragm and of the portal vein wall; moderate (score 2), in the case of a moderate increase of liver echogenicity with slightly impaired appearance of the portal vein wall and the diaphragm; severe (score 3), in the case of marked increase of liver echogenicity with poor or no visualization of portal vein wall, diaphragm, and the posterior part of the right liver lobe [27].

### 2.3. Anthropometric Measurements on Body Composition, Adiposity Distribution and Obesity

The study subjects underwent anthropometry measures using the BC300 whole-body and segmental Body Composition Analyzer (ACCUNIQ BC300, SELVAS Healthcare, Seoul, Republic of Korea). BMI was calculated as body weight (kg) divided by height (m) squared. Bioelectrical impedance was used to measure whole-body fat and lean tissue mass, and general adiposity was expressed as a percentage body fat. Abdominal adiposity was measured using waist/hip circumference (WHC). Visceral fat tissue area (cm^2^) against total body adiposity was expressed as percentage visceral adiposity grade. OB was defined as BMI ≥30 kg/m^2^ or percentage body fat >25% for men and >32% for women. Central OB was defined as WHC of >0.9 for men and >0.85 for women and VOB as percentage visceral fat >10%, according to the World Health Organization [28].

### 2.4. Assessment of Habitual Dietary Intakes of Choline, Betaine and Folate by Quantitative Food Frequency Questionnaire (qFFQ)

To assess the participants’ habitual dietary MDN, we constructed a specialized quantitative food frequency questionnaire (qFFQ) specifically designed for the assessment of choline, folate, and betaine with reference to the previously described semiquantitative FFQ for folate [29] and for choline [30]. This specialized qFFQ-MDN included 150 food items with a high nutrient density of folate, choline, and betaine, comprising 20 staple foods, 59 vegetables, 26 fruits, 38 meat and dairy products, 11 soybean products, and 9 types of nuts and fats. The food list in the qFFQ-MDN contained the 50 most frequently consumed food items for macronutrients or micronutrients reported by the Nutrition and Health Survey in Taiwan (NAHSIT) [31], and the most frequently consumed food items for choline and betaine in Taiwan [32].

Registered dietitians conducted one-on-one in-person interviews with all participants to complete the qFFQ-MDN by recording the consumption frequency of one standard serving of a specific food item from the five categories in the past year (frequency of consumption per day, week, month, and year or never consumed) and the consumption of standard servings for each food item by using visual aids comprising of measuring cups and spoons. The standard serving sizes provided with the qFFQ were based on a typical or natural portion size consumed in Taiwan [31,32]. For lipid intake measurement, the dietitians assessed the consumed portion size against a food composition table for calculating standard oil usage for preparing a meal based on a reference cooking procedure.

The current food composition database in Taiwan does not contain choline and betaine values. Therefore, we constructed the food composition data bank of choline and betaine content for the food items in the qFFQ-MDN by integrating the values from the U.S. Department of Agriculture (USDA) database [33] with other scientific analytical reports [34]. Identical food items in the aforementioned database were matched to those in the qFFQ-MDN and NAHSIT databases to assign choline and betaine values. Values for non-identical food items were determined on the basis of comparable food items. Software programming was used to construct an analytical network and app platform to integrate the choline/betaine data bank with national food composition data and generate a computerized nutrition database for analyzing total energy intake, macronutrient (proteins, lipids, carbohydrates, and fiber) intake, and intake of 33 micronutrients including folate. The qFFQ-MDN, choline/betaine data bank, computerized nutrition database, analytical network, and app platform were registered for Fu Jen Catholic University Medical Research (FJCUMR) and maintained in the Biomedicine Core Research Lab at FJCU.

### 2.5. Blood Metabolic Markers Measurements

Within 1 week of the HS diagnosis, 12 h fasting blood samples were collected in an EDTA container, maintained on ice, and transported to the FJCUMR laboratory within 2 h. Upon arrival, the EDTA-plasma were immediately separated and stored at −80 °C until further analysis. Plasma aspartate transaminase (AST) and alanine transaminase (ALT) concentrations were measured according to standard protocols (ITC Diagnostics, Taipei, Taiwan). Blood glucose, total cholesterol, TG and insulin levels were determined with enzyme-linked immunosorbent assay (Mercodia, Uppsala, Sweden) by using a Hitachi 911 analyzer. Insulin resistance was estimated using the homeostatic model assessment of insulin resistance (HOMA-IR) by standard protocols and calculation (glucose (mmol) × insulin (μU/mL)/22.5) [35]. Plasma folate levels were measured using radioimmunoassay kits (Becton Dickinson, Franklin Lakes, NJ, USA). Total homocysteine (Hcy) levels in plasma were measured using a commercially available kit for fluorescence polarization immunoassay on an Abbott 130 AxSYM system (Becton Dickinson). Plasma concentrations of betaine and free choline were measured using liquid chromatography/electrospray ionization–isotope dilution mass spectrometry, as described previously [36].

### 2.6. Statistical Analysis

Statistical analyses were performed using STATA version 13 (SAS Institute, Cary, NC, USA). Continuous variables were compared using the nonparametric Kruskal–Wallis test, and categorical variables, using the chi-square test. Categorical variables were compared using the chi-square test. The MDN and macronutrient intake, and clinical metabolic markers were calculated within strata of HS, body adiposity-defined obesity by analysis of covariance. Dependence between MDN intakes, blood MDN biomarkers, and body adiposity distribution was evaluated using Spearman correlation coefficient in all participants and HS-stratified subgroups. Multiple linear regression analyses were used to identify determinants of HS and obesity according to quartile MDN intake levels. Logistic regression models were used to examine the interactive associations between threshold intakes of MDN, MDN intake composition, and risk of HS. Explanatory HS prediction variables (age, sex, energy intake, fiber, BMI, obesity indexes, blood lipids, glucose, and HOMA-IR) were inputted in various multiple-adjusted models to control their possible confounding effects. The relationship between a given parameter and HS was calculated using the odds ratio (OR) and 95% confidence interval (CI) and two-sided *p* value. *p* values for trend and interaction were analyzed with the Cochran–Armitage linear trend test. Differences were considered statistically significant at *p* < 0.05.

## 3. Results

### 3.1. Basic Data, Body Adiposity and Blood Metabolic Markers of the Study Participants

Clinicodemographic characteristics of the study patients are presented in Table 1. The median age of the HS group (58 years) was lower than that of the control group (63 years) (*p* < 0.002). Compared with the controls, the HS group had significantly higher BMI, lower lean body mass, higher total, abdominal, and visceral adiposity, and higher levels of blood metabolic markers, including triglyceride, glucose, insulin, HOMA-IR index, and ALT (all *p* < 0.001). Sex distribution and lifestyle factors, including smoking and drinking habits, did not differ between the groups

### 3.2. Individual Methyl-Donor Nutrients (MDNs) Intake of the HS and Obesity (OB)-Stratified Participants

**Figure 1:**
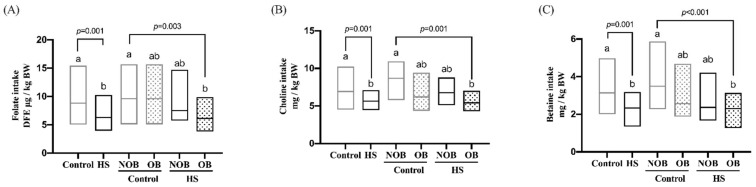
Individual methyl-donor nutrients (MDNs) intake of the HS and obesity (OB)-stratified participants. The habitual intake of folate (**A**), choline (**B**) and betaine (**C**) in the HS and OB-stratified subjects was assessed by quantitative food frequency questionnaire (qFFQ). OB was defined by BMI (≥30 kg/m^2^), waist/hip circumference ratio (men ≥ 0.9 and female ≥ 0.85), or visceral adiposity grade (>10) or percentage body fat (men ≥ 25% and women ≥ 32%). All intakes are expressed as per kg body weight values. The data are presented as medians and interquartile range (25th, 75th). Variables were compared using the non-parametric test of Kruskal–Wallis. Values with different letter differ significantly at *p* < 0.05. Abbreviations: BW, body weight; DFE, dietary folate equivalent; NOB, non-obesity.

The habitual MDN intake of the study participants was assessed using the qFFQ, and the data are presented in Supplemental Table S1. The average folate intake of the HS group was marginally lower than that of the controls (555 vs. 671 dietary folate equivalent (DFE) μg /day, *p* = 0.05), with an insufficient intake rate of 27–30% for both groups. The average choline intake of the HS (430 mg/day) and control (460 mg/day) groups did not differ, with an insufficient intake rate of 21–25%. The average betaine intake of the HS group (183 mg/day) was significantly lower than that of the control group (233 mg/day), with an insufficient intake rate of 21–39%. Because of the wide range of body weight (BW) and BMI differences between the control and HS groups, MDN intake was normalized to BW values. As illustrated in Figure 1, the BW-normalized intake of folate, choline, and betaine in the HS group was lower than that of the control group. The HS group with OB had lower BW-normalized intake of folate (*p* = 0.003), choline (*p* < 0.001), and betaine (*p* = 0.001) than the control group without OB. Dietary macronutrient intakes of the subgroups are summarized in Supplemental Table S2. Total carbohydrate, lipid, and protein intake did not differ between the HS and control groups, regardless of stratification by OB. The HS with OB group had a lower fiber intake than the control group without OB (*p* = 0.03).

### 3.3. Correlation of Blood Marker-Validated MDNs Intake with Body Fat Distribution

**Figure 2:**
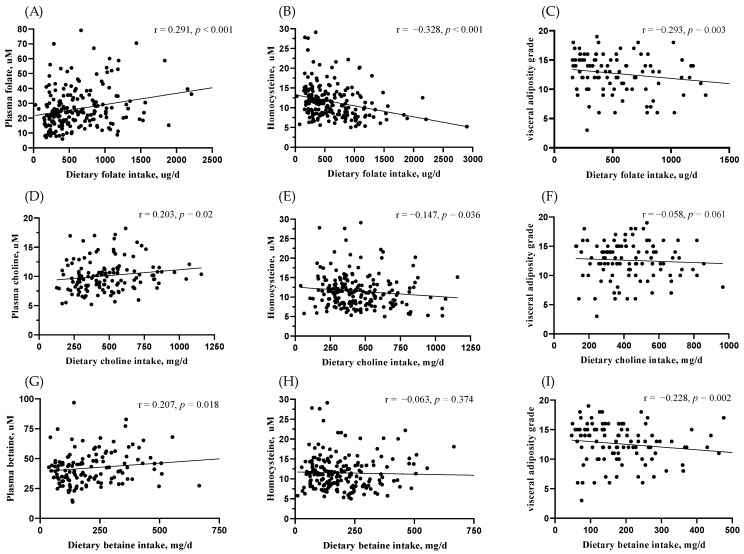
Association of MDNs intake with their blood markers and visceral adiposity. Association of MDN intake with their blood biochemical markers and Hcy levels for folate (**A**,**B**), choline (**D**,**E**) and betaine (**G**,**H**) in the total study subjects was analyzed by spearmen coefficient. Spearmen correlation of MDNs intake with visceral adiposity for folate (**C**), choline (**F**) and betaine (**I**) was analyzed in the HS group. Differences were considered to be statistically significant at *p* < 0.05.

The qFFQ-estimated MDN intakes were validated by their respective blood markers (Figure 2). Results from this validation revealed a significant positive association of MDNs intake with the blood biochemical status for folate (r = 0.29, *p* < 0.001), betaine (r = 0.20, *p* = 0.02), and choline (r = 0.20, *p* = 0.01). Plasma Hcy levels, the functional blood marker of MDN metabolism, were inversely associated with folate (r = −0.32, *p* < 0.001) and choline intake (r = −0.14, *p* = 0.03). Among various body adiposity indexes, only visceral adiposity was inversely associated with folate (r = −0.29, *p* = 0.003) and betaine intake (r = −0.22, *p* = 0.002) in the HS group (Figure 2C,I), but not in the control group (Supplemental Table S3).

### 3.4. Potential Dietary and Blood Determinants of HS in the Total and Visceral Obesity (VOB)-Stratified Participants

The dietary and blood determinants of HS in the total and VOB–stratified participants were evaluated using multivariable linear regression (Table 2). After adjustment for age, sex, and BMI, dietary HS determinants in the total participants were folate intake (β: 0.20, *p* = 0.02) and choline intake (β: −0.22, *p* = 0.05). Among the participants with VOB, choline intake was the most significant dietary determinant of HS in that a single-unit increase in choline intake was associated with a 0.41 decrease in hepatic lipid accumulation (β: −0.41, *p* = 0.01). Such a significant inverse association of choline intake with HS was not observed in participants without VOB. Macronutrient intake (lipid, carbohydrate, protein, and fiber) and energy consumption were not associated with HS. Blood triglyceride levels (β: 0.32, *p* < 0.001) and insulin resistance (β: 0.29, *p* = 0.004) were the two most significant clinical determinants of HS in the subjects with VOB. Insulin resistance was the most significant HS determinant (β: 0.54, *p* < 0.001) in the participants without VOB. Blood Hcy concentration was not correlated with HS.

### 3.5. Quartile Intake of Individual MDN Associated with HS and VOB

The threshold intake of individual MDNs associated with HS and VOB was investigated using multivariable regression models (Table 3). Total choline intake was divided into quartiles (Q1–Q4), with Q1 intake considered the deficiency level. The data indicated that the choline intake at Q4 versus Q1 was inversely associated with HS (OR: 0.32, 95% CI: 0.11–0.97) and VOB (OR: 0.28, 95% CI: 0.08–0.95) after adjustment for age, sex, BMI, waist/height (W/H) circumference ratio, blood hypertriglyceridemia, and insulin resistance (Model B). Folate intake was also divided into four levels (Q1–Q4), with Q1 intake considered the deficiency level. The folate intake at Q4 versus Q1 was inversely associated with HS (OR: 0.36, 95% CI: 0.13–0.98) and VOB (OR: 0.25, 95% CI: 0.07–0.89) after adjustment for age, sex, BMI, and W/H ratio (Model A). Further adjustment for blood triglyceride levels and insulin resistance negated the Q4 folate intake effect on both outcomes (data not shown). Q4 versus Q1 betaine intake was inversely associated with HS (OR: 0.31, 95% CI: 0.12–0.85) and VOB (OR: 0.26, 95% CI: 0.08–0.89) (Model A). Further adjustment for blood triglyceride levels and insulin resistance negated the betaine intake–HS and VOB association (data not shown).

### 3.6. Threshold Intakes of Individual MDN Associated with VOB-Related HS

We determined threshold MDN intake at the cutoff level of median BW-normalized intake of the controls with VOB-related HS (Table 4). Among participants with low choline intake, those with VOB had a 22-fold increased OR of HS (95% CI: 8.0–6.1) compared with those without VOB, after adjustment for age, sex, and energy and fiber intake (Model A: OR: 22, 95% CI: 8.1–63). This effect on VOB-related HS risk was decreased two-fold by high choline consumption (Model A: OR: 10, 95% CI: 2.9–37). Further adjustment for blood metabolic markers (triglyceride, cholesterol, HOMA-IR, and HbA1c) did not alter the VOB–choline intake relationship in HS (Model B). Among participants with low betaine intake, those with VOB had a 26-fold increased OR of HS (Model A: OR: 26, 95% CI: 9.4–75) compared with those without VOB, and high betaine intake was associated with a two-fold reduced HS after adjustment for age, sex, and energy and fiber intake (Model A: OR: 12, 95% CI: 3.3–45). Further adjustment for blood metabolic markers did not alter the VOB–choline intake relationship in HS (Model B). Among participants with low and high folate intake, those with VOB had a 19-fold (Model B: OR: 19, 95%: 5.2–74) and 30-fold increased OR of HS (Model B: OR: 30, 95% CI: 5.7–156), respectively, compared with those without VOB.

### 3.7. Combined MDNs Intake Composition Associated with VOB-Related HS

Finally, we explored the optimal MDN intake composition for minimizing obesity-related HS (Table 5). Individuals with high folate and betaine intake at the designated cutoff had 67% reduced OR of HS (OR: 0.33, 95% CI: 0.14–0.75), independent of age and sex (Model 1: OR: 0.33, 95% CI: 0.14–0.75). Adjustment for VOB (Model 2) reversed and negated such a significant high FA/CH intake–HS relationship (Model 2). Further adjustment for blood metabolic disorders and blood one-carbon biomarkers did not alter the VOB-modifying HS (Models 3 and 4). Individuals with high folate and choline intake had a 58% reduced OR of HS (OR: 0.42, 95% CI: 0.19–0.84), independent of age and sex (Model 1). Adjustment for VOB (Model 2) negated such a significant high FA/CH intake–HS relationship (Model 2). Further adjustment for blood metabolic disorders did not alter the VOB-modifying HS (Models 3 and 4). Combined high betaine and choline intake composition was associated with an 81% reduced OR of HS, independent of all tested HS risk factors (Model 4: OR: 0.19, 95% CI: 0.05–0.69). Adding high folate intake to this choline and betaine intake composition negated the relationship (Model 2: OR: 0.37, 95% CI: 0.12–1.14).

## 4. Discussion

To the best of our knowledge, this study is the first to comprehensively examine the relationship between MDN intake and obesity-related HS. In relation to folate and betaine, total choline intake was identified as the most significant dietary determinant of VOB-related HS after adjustment for the major contributors to HS in participants with obesity [4,5,6,37]. Our findings on the inverse association of choline intake with VOB and HS are consistent with previous studies involving 3214 Canadian adults [38] and 56,000 Chinese adults [21]. However, the opposite association has also been reported in a US study involving 7074 adults [39]. Such diverse effects of choline on body adiposity and HS may be attributable to sex and age [40], dietary energy [41] and fiber [42,43] intake, and clustering metabolic disorders [37]; however, our observed choline effect was independent of those confounding factors. The threshold choline intake for minimizing VOB-related HS remains unclear. A low-choline diet (<50 mg/day) can cause HS in healthy adults [44]. In 644 patients with NAFLD, low choline intake of less than 50% of the defined US AI (212 mg/day) was associated with the progression of steatosis to fibrosis in postmenopausal women [22]. High choline intake was associated with better body adiposity distribution in a Canadian adult population (mean choline intake: 313 mg/day) [38] and NAFL reduction in Chinese women of normal weight (mean choline intake: 412 mg/day) [21]. Similarly, in our study, a favorable threshold choline intake of adult AI value at the Q4 level was associated with a reduction in HS and visceral adiposity. In particular, for participants with VOB, the most susceptible subgroup in terms of HS development [5,6,7], choline intake below the adult AI value was associated with a 22-fold increased OR of HS compared with those without VOB. Increasing choline intake in the participants with VOB above the favorable threshold choline intake was associated with a two-fold decreased OR of VOB-related HS; however, this did not overcome the residual effect of VOB’s promotion of HS. Our data suggest that choline intake plays a central role in VOB-related HS.

Compared with choline intake, individual folate or betaine intake exhibited a similar inverse but weaker association with visceral adiposity and HS in this and the previous study [38]. Our findings on the inverse association of folate and betaine intake with visceral adiposity but not with other adiposity measures (BMI, WHC, and total body fat) are in line with the findings of a study on 175 overweight young adults in which the consumption of non-starch vegetables and dark green or bright orange/yellow vegetable intake, rich in folate and betaine, was associated with lower liver fat deposition and visceral adiposity [45]. A 2-year longitudinal study on 624 healthy Japanese adults found an inverse association of folate intake with visceral adiposity but not with BMI [46]. In 109 overweight young adults, folate intake was not associated with computed tomography–measured visceral adiposity tissue and subcutaneous fat [47]. Threshold folate intake at T2/T3 levels (407–511 μg) was associated with decreased BMI-defined obesity rate among 2695 healthy adults [48], whereas high folate (1192 DFE ug/day) and betaine intake (Q4: 407 mg/day) was associated with reduced abnormal hepatic and visceral fat accumulation in our study patients with metabolic disorders. After adjustment for VOB modifiers such as dietary fiber and energy intake [41,42,43] and VOB–linked blood metabolic orders such as TG, cholesterol, insulin resistance, and HbA1c, low folate and betaine intake below the Q4 levels predicted 14–19 fold increased OR of HS in patients with VOB compared with those without VOB. Individual high folate and betaine intake did not overcome the residual effect of VOB on HS. The high folate–obesity effect on HS was amplified after adjustment for hypertriglyceridemia and insulin resistance, suggesting their mediating role in folate and obesity-related HS. Consistently, high blood folate status independently predicted altered insulin resistance and NALFD-related metabolic syndrome in 278 obesity patients [49].

A novel finding in our study is the identification of the MDN intake composition of choline and betaine that can minimize VOB-related HS. Combined high choline and betaine intake was associated with an 81% reduced OR of HS after adjustment for VOB and VOB-related metabolic disorders. However, the mechanism underlying this association remains elusive. Multiple hypotheses are plausible. In animals fed with a high-fat diet to induce obesity, hepatic lipid accumulation was associated with poor TG-containing lipoprotein secretion, which places increased demand on choline for PC synthesis for its lipotropic effect [15,50]. Under obesity-induced excess fat accumulation, the increased requirement for PC synthesis may rely on hepatic salvage PC synthesis by the PEMT pathway using folate and betaine-mediated S-adenosylmethionine-derived methyl groups. Depletion of folate and betaine in mice with high fat diet-induced obesity caused secondary depletion of choline and PC in rat liver and hyperhomocysteinemia, which resulted in higher oxidative stress and metabolic dysfunction such as insulin resistance [51]. However, choline supplementation alone did not reduce liver steatosis of PEMT^-/-^ mice fed a high-fat diet [52]. In choline-feeding studies involving Mexican-American men [53] and healthy men [54,55], total choline intake exceeding AI levels was reported to optimize PC and free choline levels in blood and the liver, thus reducing liver dysfunction markers. High betaine supplementation could substitute for choline and folate to normalize Hcy levels under methyl donor methionine-restriction conditions [56]. Preformed betaine intake from whole-grain foods and vegetables can lower the obesity-increased choline and folate requirements by sparing choline oxidation for betaine synthesis and folate for methyl donor conversion in one-carbon metabolism. Impaired one-carbon metabolism in BHMT-knockdown mice disrupted the epigenetic regulation of hepatic TG accumulation with altered choline and PC levels and reduced very-low-density lipoprotein secretion [15,57]. Administration of betaine to high-fat diet–fed rats modulated folate/choline metabolism and protected the liver from oxidative stress and steatosis [58]. These mechanisms may at least partly explain the synergistic effect of high choline and betaine in minimizing VOB-related HS OR in the present study.

The metabolic activity of this optimal choline and betaine intake composition for VOB-related HS may not be limited to hepatic lipid homeostasis. The effect of dietary intake on energy metabolism in adipocytes may also play a role. In animal studies, genetic defects in *CTP:phosphoethanolamine cytidylyltransferase* (*Pcyt2*), a key enzyme of hepatic PC synthesis, led to obesity and obesity-related HS development [59]. One month of choline supplementation facilitated triacylglyceride degradation in Pcyt2^+/−^ adipocytes and in the liver [59]. High betaine intake for 8 weeks increased lipid breakdown in adipocytes, which was attributed to increased systemic energy metabolism and reduced hepatic lipid accumulation [60]. In Pcyt2-deficient mice, combined choline and betaine supplementation modulated mitochondrial oxidative demethylation, thus improving lipid homeostasis in adipose tissues and in the liver [19]. By contrast, compared with wild-type mice, *Pemt*^+/−^ mice were protected from high-fat diet–induced obesity and insulin resistance, and this protection was negated by choline supplementation [61]. However, in these genetically defective mice models, how dietary choline and betaine intake affects cross talk in energy and lipid metabolism between adipocytes and hepatocytes to modulate liver fatty development remains unclear. In the present study, choline and betaine intake did not significantly interact with VOB for HS modulation. The effect of choline and betaine composition on lower HS-OR remained significant after adjustment for VOB and metabolic dysfunction markers of hypertriglyceridemia (indicating abnormal hepatic and systemic lipid metabolism) and insulin resistance (indicating hepatic energy metabolism regulation). Future studies should identify the gene polymorphic contributing factors that might modify hepatic and visceral fat accumulation by this optimal MDN composition.

Notably, additional increases in folate intake above three-fold normal levels counteracted the effect of the combined choline and betaine intake composition on VOB-related HS, increasing lipid storage, weight gain, and adipose tissue inflammation in rats [62]. High folate consumption leads to pseudo-MTHFR deficiency, altered lipid metabolism, and liver injury in mice [63]. Consistently, we observed that a further increase in folate intake counteracted the anti-VOB related HS effect of the combined choline and betaine composition. The interconnection of bioenergetic and one-carbon methylation metabolism with the interwoven metabolic pathways of choline, folate, and betaine further complicates their individual or mutual requirement for protection against obesity-related HS [45]. Future studies should investigate counteracting molecular targets of high folate intake with respect to the combined effect of choline and betaine on reducing HS and obesity risks.

## 5. Limitations and Strengths of the Study

The current study has several limitations. The dietary intake history of participants was recall based, requiring selection from a predefined foods list with an estimated intake frequency; this may have led to an underestimation of the intake, in particular for macronutrients, resulting in a null difference in energy intake between obese and non-obese subgroups. Furthermore, the choline and betaine intake levels of the participants were analyzed according to the hypothetical food composition data of those two nutrients constructed from the USDA databank, which may not accurately capture the dietary choline and betaine intake from the local food items. Analytical values of choline and betaine of frequently consumed food items in our FFQ are required. Another limitation is that we did not measure plasma PC with the other lipids in the study. Despite dietary choline intake being strongly associated with plasma-free choline levels, we did not measure plasma PC in the study. Dietary choline quantitatively contributes to blood PC, a constitutive compound highly concentrated in livers and in all lipoproteins. HS and OB-associated changes of blood PC may reflect how dietary choline modifies hepatic lipid metabolism in these subgroups, which warrants future analysis. The other limitation of this study is to use many subdivisions for analysis of VOB-related HS associated with three NDM intakes. When our study groups contain a small number of people, the “risk estimates” becomes unstable. The major limitation is the use of a case–control study design which does not establish a causal relationship. Direct intervention studies with reasonable sample sizes and well-matched subjects are required to confirm the optimal choline and betaine intake composition for minimizing obesity-related HS. Although multiple well-established risk factors were comprehensively adjusted in our analysis, genetic factors influencing MDNs interwoven metabolic pathways such as BHMT and PEMT variations were not completely ruled out, which are the aims of our continued study.

The strengths of this study include the investigation of obesity-related HS in the setting of a high-risk population with a high burden of metabolic disorders. Blood marker–validated MDN intake from the qFFQ was effective for examining the association of habitual intake with HS, and the results indicated a close metabolic link. Dietary recommendations remain the cornerstone of NAFL treatment, and to the best of our knowledge, this study is the first to identify the optimal dietary MDN intake composition for minimizing obesity-related NAFL risks. Finally, our study calls for detailed MDN nutrient documentation, rather than a single supplement, in future trials, especially given their interwoven relationship in influencing disease progression.

## 6. Conclusions

In summary, our data suggest that combined dietary intake of choline and betaine reduces the VOB-related HS risk in a threshold-dependent manner. These data provide fundamental insight into the complexity of the protective effects of the optimal dietary MDN intake composition and VOB-related HS in populations with a high prevalence of VOB, such as in Asia. More comprehensive trials and experiments are necessary to characterize genetic and molecular targets in the pathophysiology of obesity-related impairment in hepatic lipid metabolism for enhanced nutritional prevention and intervention, in order to maximize the HS risk reduction through optimization of MDN intake composition.

## Figures and Tables

**Table 1:** Basic and clinical data of the study participants ^1,2^.

Variables	Control	HS	*p* Value
Sex			
Men, n (%)	47 (45)	46 (44)	0.84
Age, y	63 (55, 68)	58 (46, 65)	0.002
BMI, kg/m^2^	22 (20, 24)	27 (24, 29)	<0.001
<18.5, n (%)	9 (9)	1 (1)	<0.001
18.5–24, n (%)	63 (61)	17 (16)	
24–27, n (%)	20 (19)	34 (32)	
≥27, n (%)	12 (11)	53 (51)	
Body composition			
Skeletal muscle mass ^3^, %	39 (35, 43)	37 (34, 39)	0.004
Body fat ^4^, %	28 (21, 33)	32 (28, 36)	<0.001
Waist/hip circumference	0.8 (0.8, 0.9)	0.9 (0.9, 1.0)	<0.001
Visceral adiposity grade	6 (5, 9)	13 (10, 15)	<0.001
Smoking status			
Current and former smoker, n (%)	17 (16)	23 (22)	0.36
Alcohol use			
Current and former, n (%)	16 (15)	19 (18)	0.49
Blood biochemical marker			
Triglycerides, mg/dL	72 (53, 95)	135 (97, 187)	<0.001
Total cholesterol, mg/dL	197 (170, 224)	191 (173, 215)	0.44
Plasma glucose, mg/dL	98 (94, 104)	104 (98, 116)	<0.001
Insulin, μIU/ml	7.9 (6.4, 10)	13 (10, 19)	<0.001
HOMA-IR	1.9 (1.6, 2.7)	3.7 (2.5, 5.7)	<0.001
AST, U/L	23 (19, 26)	20 (17, 28)	0.12
ALT, U/L.	19 (16, 25)	24 (16, 38)	0.04

^1^ Sample size for the control and hepatic steatosis (HS) groups are 104 and 105, respectively. ^2^ Continuous variables are presented as medians and interquartile ranges (25th, 75th). Values were compared using the non-parametric test of Kruskal–Wallis. Discrete variable was expressed as numbers with proportions in parenthesis. Values were compared using the chi square test. Significance is defined at *p* < 0.05. ^3^ Medians and interquartile ranges (25th, 75th) for skeletal muscle mass by kg is 21 (18, 27) for controls and 27 (22, 31) for HS. ^4^ Medians and interquartile ranges (25th, 75th) for body fat by kg is 16 (12, 20) for controls and 22 (19, 26) for HS. Abbreviations: ALT, alanine transaminase; AST, asparte transaminase; BMI, body mass index; HOMA-IR, homeostatic model assessment of insulin resistance.

**Table 2:** Potential dietary and blood determinants of HS in the total and VOB-stratified participants ^1,2^.

Independent Variables	Dependent Variables of Hepatic Steatosis					
	Total Subjects		VOB		Non-VOB	
	β	*p* Value	β	*p* Value	β	*p* Value
Dietary intakes						
Folate, DFE μg/day	0.20	0.02 *	0.24	0.07	0.09	0.5
Choline, mg/day	−0.22	0.05	-0.41	0.01 *	0.05	0.8
Betaine, mg/day	-0.04	0.6	-0.01	0.9	−0.13	0.4
Lipid, g/day	0.14	0.1	0.18	0.1	−0.20	0.5
Carbohydrate, g/day	0.11	0.3	0.12	0.4	−0.25	0.7
Energy, Kcal/day	−0.17	0.3	-0.04	0.8	0.27	0.8
Fiber, g/day	−0.07	0.4	-0.13	0.5	0.01	0.9
Blood metabolic markers						
Triglycerides, mg/dL	0.28	<0.001 ***	0.32	<0.001 ***	0.15	0.09
HOMA-IR index	0.28	<0.001 ***	0.29	0.004 **	0.54	<0.001 ***
Homocysteine, uM	0.06	0.2	0.01	0.8	-0.04	0.6

^1^ Linear regression models were constructed to evaluate the determinants of HS (sonography-graded fatty liver: 0 = no, 1 = mild; ≥2 = moderate/sever) in the total and visceral obesity (VOB)-stratified subgroups (visceral adiposity grade > 10). Differences were considered to be statistically significant at * *p* < 0.05, ** *p* < 0.01 and *** *p* < 0.001. ^2^ Multivariables linear regression models were adjusted for age, sex and BMI. Abbreviations: DFE, dietary folate equivalent.

**Table 3:** Quartile intake of individual MDN associated with HS and VOB ^1,2^.

Quartile MDNs Intake	HS	VOB
Choline intake mg/kg body weight	Model B odds ratio (95% CI)	Model B odds ratio (95% CI)
Q1: 3.7 (3.2, 4.1)	1 (ref.)	1 (ref.)
Q2: 5.2 (4.7, 5.7)	1.3 (0.49–3.95)	0.54 (0.16–1.8)
Q3: 7.0 (6.5, 7.5)	1.1 (0.40–3.29)	0.85 (0.25–2.8)
Q4: 10 (9.6, 13)	0.32 * (0.11–0.97)	0.28 * (0.08–0.95)
Folate intake DFE μg/kg body weight	Model A odds ratio (95% CI)	Model A odds ratio (95% CI)
Q1: 3.3 (2.6, 3.9)	1 (ref.)	1 (ref.)
Q2: 5.7 (5.0, 6.5)	0.96 (0.36–2.62)	0.83 (0.23–3.0)
Q3: 9.6 (8.1, 11)	0.82 (0.30–2.20)	0.83 (0.23–3.0)
Q4: 17 (14, 21)	0.36 * (0.13–0.98)	0.25 * (0.07–0.89)
Betaine intake mg/kg body weight	Model A odds ratio (95% CI)	Model A odds ratio (95% CI)
Q1: 1.1 (0.91, 1.3)	1 (ref.)	1 (ref.)
Q2: 2.1 (1.9, 2.3)	0.79 (0.30–2.0)	0.89 (0.26–3.0)
Q3: 3.1 (2.9, 3.6)	0.86 (0.32–2.2)	0.76 (0.22–2.6)
Q4: 5.6 (4.7, 6.9)	0.31 * (0.12–0.85)	0.26 * (0.08–0.89)

^1^ Multivariable logistic regression models were constructed to evaluate quartile intake of individual MDN associated with hepatic steatosis and visceral fat accumulation. Q1 intake was used for reference. VOB was defined by visceral adiposity grade (> 10) for prediction variable. The values were expressed as median and interquartile range (25th, 75th). ^2^ Multivariable adjusted models: Model A: adjusted for age, sex, BMI, and W/H circumference. Model B: Model A was additionally adjusted for hypertriglyceridemia (TG > 150 mg/dL) and insulin resistance (HOMA-IR > 2). * Differences were considered to be statistically significant at *p* < 0.05.

**Table 4:** Threshold intake of individual MDN associated with VOB-related HS ^1,2^.

Visceral Obesity	Choline Intake ^3^ mg/kg Body Weight		Betaine Intake ^4^ mg/kg BW		Folate Intake ^5^ DFE ug/kg BW	
	Low <6.9	High ≥6.9	Low <3.1	High ≥3.1	Low <8.8	High ≥8.8
Visceral adiposity grade						
<10, n	47	51	46	52	46	50
≥10, n	76	33	80	29	73	36
Model A: OR (95%CI)						
<0 (ref.)	1 (ref.)	0.85 (0.2–3.1)	1 (ref.)	1.2 (0.3–4.3)	1 (ref.)	1.08 (0.3–3.8)
≥10	22 * (8.1–63)	10 * (2.9–37)	26 * (9.4–75)	12 * (3.3–45)	26 * (8.9–76)	13 * (3.9–48)
Model B: OR (95%CI)						
<10 (ref.)	1 (ref.)	1.33 (0.2–7.2)	1 (ref.)	1.0 (0.21–5.5)	1 (ref.)	2.6 (0.5–13)
≥10	22 * (6.5–80)	9.6 * (1.9–46)	14 * (4.4–50)	14 * (2.8–70)	19 * (5.2–74)	30 * (5.7–156)

^1^ Logistic regression models were constructed to evaluate threshold intake of MDN associated with visceral obesity (VOB)-related hepatic steatosis (HS). VOB was defined by visceral fat grade ≥10. Hepatic steatosis was diagnosed by sonography-graded fatty liver (yes or no). ORs of hepatic steatosis were considered to be statistically significant in relation to reference OR of 1 at * *p* < 0.05. ^2^ Multivariable logistic regression models were Model A: adjusted for age, sex, energy and fiber intake; Model B: additional adjustment of model A on blood triglyceride, blood cholesterol, HOMA-IR, and HbA1c. ^3^ Choline intake was stratified into the low (median 323; IQR: 244, 405 mg/d) and high intake (median: 603; IQR: 504, 720 mg/d) group at cutoff value of the controls’ median intake (6.9 mg/kg body weight = 406 mg/day). ^4^ Betaine intake was stratified into low (median 124; IQR: 82, 161 mg/d) and high intake (median 289; IQR: 233, 386 mg/d) at cutoff value of the controls’ median intake (3.1 mg/kg body weight = 178 mg/day). ^5^ Folate intake was stratified into low (median 320; IQR: 234, 421 DFE ug/d) and high intake (median 891; IQR: 289, 1211 DFE ug/d) at cutoff value of median intake of the control (8.8 ug/kg body weight = 527 DFE ug/day).

**Table 5:** Association of combined MDN intake composition with VOB-related HS risk ^1,2^.

MDN Intake Composition				Fatty Liver		Mode 1 OR (95%CI)	Model 2 OR (95%CI)	Model 3 OR (95%CI)	Model 4 OR (95%CI)
				No	Yes				
Dietary Folate ^3^ (DFE ug/d) X Betaine ^4^ (mg/d) intake									
Folate		Betaine							
Low		Low		62	76	1 (ref.)	1 (ref.)	1 (ref.)	1 (ref.)
Low		High		5	4	0.46 (0.1–2.06)	0.93 (0.14–6.08)	0.55 (0.05–5.41)	0.59 (0.07–5.16)
High		Low		12	13	0.69 (0.28–1.72)	1.32 (0.39–4.49)	1.88 (0.47–7.55)	2.10 (0.50–8.82)
High		High		24	11	0.33 * (0.14–0.75)	0.42 (0.15–1.21)	0.42 (0.14–1.29)	0.46 (0.14–1.54)
Dietary Folate ^3^ (DFE ug/d) X Choline ^5^ (mg/d) intake									
Folate		Choline							
Low		Low		46	53	1 (ref.)	1 (ref.)	1 (ref.)	1 (ref.)
Low		High		21	27	0.86 (0.39–1.85)	0.67 (0.24–1.87)	0.47 (0.14–1.56)	0.4 (0.14–1.51)
High		Low		5	4	0.58 (0.14–2.41)	2.58 (0.45–14.9)	4.18 (0.59–29.2)	4.14 (0.51–33.3)
High		High		31	20	0.42 * (0.19–0.89)	0.44 (0.16–1.17)	0.41 (0.14–1.21)	0.53 (0.17–1.64)
Dietary Betaine ^4^ (mg/d) X Choline ^5^ (mg/d) intake									
Betaine		Choline							
Low		Low		48	56	1 (ref.)	1 (ref.)	1 (ref.)	1 (ref.)
Low		High		27	34	0.89 (0.45–1.79)	0.58 (0.23–1.46)	0.37 (0.12–1.14)	0.32 (0.10–1.0)
High		Low		4	2	0.51 (0.08–2.95)	0.88 (0.11–7.11)	0.33 (0.02–4.73)	0.24 (0.02–3.57)
High		High		25	13	0.33 * (0.14–0.78)	0.31 * (0.10–0.92)	0.26 * (0.08–0.84)	0.19 * (0.05–0.69)
Dietary Choline ^5^ (mg/d) X Betaine ^4^ (mg/d) X Folate ^3^ (DFE ug/d) intake									
Choline	Betaine		Folate						
Low	Low		Low	43	51	1 (ref.)	1 (ref.)	1 (ref.)	1 (ref.)
One or two of the three MDN intake was low				38	43	0.76 (0.4–1.5)	0.89 (0.4–2.1)	0.71 (0.3–1.9)	0.72 (0.3–1.9)
High	High		High	23	11	0.33 * (0.1–0.8)	0.37 (0.1–1.1)	0.33 (0.1–1.1)	0.33 (0.1–1.1)

^1^ Ordinal logistic regression models were constructed to evaluate the MDN intake composition associated with hepatic steatosis risk. * ORs of hepatic steatosis were considered to be statistically significant in relation to reference OR of 1 at *p* < 0.05. ^2^ Multivariable adjust models: Model 1: adjusted for age, sex, Model 2: Mode l was additionally adjusted for visceral obesity (visceral adiposity grade ≥10), abdominal obesity (W/H circumference: men ≥ 0.9, women ≥0.85), and general obesity (BMI ≥ 30 kg/m^2^). Model 3: Model 2 was additionally adjusted for hypertriglyceridemia (TG > 150) and insulin resistance (HOMA-IR >2). Model 4: Model 3 was additionally adjusted for Hcy, blood folate, choline or betaine status. ^3^ Folate intake was stratified into low and high intake at cutoff value (Q4 levels: 781 DFE μg/day). ^4^ Betaine intake was stratified into low and high intake at cutoff value (Q4 levels: 280 mg/day). ^5^ Choline intake was stratified into low and high intake at cutoff value of current Taiwanese adult AI intake for men (450 mg/d) and for women (390 mg/day).

## Supplementary Materials

The following are available online at https://www.mdpi.com/article/10.3390/nu14020261/s1, Table S1: MDN intake of the total and obesity-stratified subjects. Table S2: Macronutrients intake of the total and obesity-stratified subjects. Table S3: Spearman correlation of MDN intake with adiposity distribution in the total and obesity-stratified subgroups.
