# Supplementary material for: Optimal Dietary Intake Composition of Choline and Betaine Is Associated with Minimized Visceral Obesity-Related Hepatic Steatosis in a Case-Control Study
Source: Nutrients. 2022 Jan 8;14(2):261. doi: 10.3390/nu14020261 (PMC8779168; doi:10.3390/nu14020261)
Supplement: Supplementary file 1 [file nutrients-14-00261-s001.zip › nutrients-1504490-supplementary.pdf]

Table S1. MDNs intakes of the HS and obesity-stratified subjects[1,2]

MDNs Intake	Control	HS	*p* Value
Folate	671 ± 480 (27)	555 ± 357 (30)	0.051
DFE μg/day,(insufficient intake, %)[3]			
Non-Obesity	712 ± 560 (28)	557 ± 288 (33)	0.205
Obesity	622 ± 401 (25)	555 ± 366 (30)	
Betaine	233 ± 182 (21)	183 ± 105 (29)	0.017
mg/day,(insufficient intake, %)[4]			
Non-Obesity	261 ± 207a (18)	174 ± 82ab (30)	0.028
Obesity	208 ± 153ab (23)	185 ± 108b (29)	
Choline	462 ± 231 (25)	430 ± 176 (21)	0.262
mg/day, (insufficient intake, %)[5]			
Non-Obesity	505 ± 248 (18)	432 ± 210 (23)	0.152
Obesity	424 ± 210 (30)	429 ± 172 (20)	

[1]Data are presented as the mean ± SD for the continuous variables and as proportion (insufficiency rate :%) for the categorical variables. Variables that were not normally distributed were first log-transformed. The continuous variables were compared using the student t test or one-way ANOVO following tukey test. Values with different letter differ significantly at P < 0.05. [2]Obesity was defined by BMI (≥30 kg/m2), waist/hip circumference ratio (men ≥ 0.9 and female ≥0.85), visceral adiposity grade (≥10) or/and body fat (men ≥25 % and women ≥ 32 %). [3]Insufficient folate intake was defined as total intake below the folate EAR value for Taiwanese adults (320 μg/day). [4]Insufficient betaine intake was defined as total intake below 25 percentile of total subject intake (112 mg/day).[5]Insufficient choline intake was defined as total intake less than 70% AI value for Taiwanese adults of men (315 mg/day) and women (273 mg/day).

Table S2. Macronutrients intake of the total subjects and obesity subgroup[1,2]

Macronutrient intake	Control (n=104)	HS (n=105)	p Value
**median (interquartile range)**			
Energy, kcal/d	1631	1513	0.19
	(1242, 2061)	(1093, 1943)	
Non-Obesity	1706	1398	0.13
	(1398, 2172)	(961, 2178)	
Obesity[3]	1544	1538	
	(1167, 1846)	(1112, 1929)	
Carbohydrate, % of energy	64 (56, 70)	63 (58, 68)	0.42
Carbohydrate, g/d	256 (189, 330)	244 (168, 302)	0.12
Non-Obesity	275	196	0.08
	(211, 340)	(149, 353)	
Obesity	236	246	
	(174, 310)	(173, 301)	
Protein, % of energy	18 (16, 21)	18 (16, 21)	0.79
Protein, g/d	75 (55, 97)	69 (50, 90)	0.28
Non-Obesity	81 (53, 115)	69 (47, 88)	0.33
Obesity	68 (56, 92)	71 (52, 91)	
Lipid, % of energy	18 (14, 23)	19 (15, 23)	0.32

Lipid, g/d	33 (23, 47)	30 (22, 43)	0.53
Non-Obesity	35 (28, 52)	27 (20, 46)	0.20
Obesity[3]	29 (21, 37)	30 (23, 43)	
Cholesterol, mg/d	344 (209, 457)	329 (232, 455)	0.93
Dietary Fiber, g/d	24 (16, 34)	19 (12, 29)	0.01
Non-Obesity	27 (17, 36) a	22 (12, 30) ab	0.03
Obesity[3]	22 (15, 33) ab	19.4 (12, 29) b	

[1]The data are presented as medians and interquartile ranges (25%, 75%). Variables were compared using the non-parametric test of Kruskal-Wallis. Values with different letter differ significantly at $P < 0.05$. [2]Obesity was defined by BMI (≥ 30 kg/m^2), waist/hip circumference ratio (men ≥ 0.9 and women ≥ 0.85), visceral adiposity grade (≥10) or/and body fat (men ≥ 25 % and women ≥ 32 %).

Table S3. Spearman correlation of MDNs intake with body adiposity index in the HS and sex-stratified subgroups[1]

Subjects	Body adiposity distribution	Folate intake		Total choline intake		Betaine intake	
		r	p	r	p	r	p
Control (n=104)	BMI	-0.068	0.497	0.032	0.746	-0.048	0.629
	Visceral fat	0.005	0.957	-0.057	0.568	-0.096	0.333
	Waist/hip circumference	-0.149	0.136	-0.085	0.394	-0.116	0.245
	Total body fat	-0.018	0.857	-0.12	0.227	-0.131	0.189
HS (n=105)	BMI	-0.106	0.285	0.091	0.354	-0.024	0.806
	Visceral fat	-0.293	0.003*	-0.058	0.061	-0.228	0.002*
	Waist/hip circumference	-0.169	0.089	0.034	0.735	-0.151	0.126
	Total body fat	-0.119	0.233	-0.136	0.17	-0.048	0.627

[1]Association of MDNs intake with adiposity distribution indexes was analyzed by spearman correlation coefficient. *Differences were considered to be statistically significant at $P < 0.05$.
